# Tracing microbial carbon sources in hydrothermal sediments by ^13^C isotopic analysis of bacterial and archaeal ribosomal RNA

**DOI:** 10.3389/fmicb.2025.1680337

**Published:** 2025-10-16

**Authors:** Barbara MacGregor, Henricus T. S. Boschker, Daniel Hoer, Daniel B. Albert, Howard Mendlovitz, Andreas Teske

**Affiliations:** ^1^Department of Earth Sciences, University of Minnesota, Minneapolis, MN, United States; ^2^Geobiology Group, Department of Biology, University of Antwerp, Wilrijk, Belgium; ^3^Earth, Marine and Environmental Sciences, University of North Carolina at Chapel Hill, Chapel Hill, NC, United States

**Keywords:** Guaymas Basin, natural ^13^C abundance, 16S rRNA, bead capture, hydrothermal carbon sources

## Abstract

Microbial communities in hydrothermal sediments of Guaymas Basin assimilate a wide range of carbon sources, detrital organic matter, DIC of hydrothermal and water column origin, as well as methane, light alkanes and petroleum hydrocarbons. Here we analyze the abundances and ^13^C-isotopic values of these carbon pools, and assess the relative importance of these carbon sources by comparison with δ^13^C-isotopic composition of bacterial and archaeal rRNA. In almost all hydrothermal sediments, δ^13^C-rRNA values for bacterial and archaea are lighter (more ^13^C-depleted) than those of TOC and DIC, indicating that carbon from ^13^C-depleted methane permeates the microbial food web, with no systematic preference for bacteria or archaea. However, the omnipresence of detrital organic matter of photosynthetic origin means that any methane signal in bacterial and archaeal δ^13^C-rRNA values is diluted by the heterotrophic background. In non-hydrothermal background sediment where methane is lacking, the δ^13^C-rRNA values for bacterial and archaea are heavier (less ^13^C-depleted) and indicate the preferential utilization of detrital TOC of photosynthetic origin. The presence of petroleum in some methane-rich hydrothermal cores does not visibly change the δ^13^C-rRNA values for bacterial and archaea, since the δ^13^C-isotopic composition of hydrothermal petroleum in Guaymas Basin is similar to its source, detrital organic carbon, and thus does not separate fossil carbon utilizers from general heterotrophs. When δ^13^C-depleted methane competes with high concentrations of δ^13^C-repleted (heavier) short-chain alkanes, δ^13^C-rRNA values for bacteria and archaea are noticeably heavier than those in methane-rich but alkane-poor sediments, suggesting short-chain alkane incorporation. Under alkane-rich conditions, consistent differences between bacterial and archaea δ^13^C-rRNA were observed, suggesting the impact of distinct bacterial and archaeal alkane assimilation pathways. To summarize, we note that the availability of different sedimentary or hydrothermal carbon sources—in particular hydrothermal methane—is reflected in changing δ^13^C-rRNA values for bacteria and archaea.

## Introduction

The hydrothermal sediments of Guaymas Basin, a young and hydrothermally active spreading center in the Gulf of California, are rich in organic substrates, DIC, methane and hydrocarbons (Peter et al., 1991; de la Lanza-Espino and Soto, 1999; McKay et al., 2016; Song et al., 2021). These compounds serve as carbon sources for diverse microbial communities, including heterotrophic bacteria (Castro et al., 2021), chemosynthetic microbial mats (MacGregor et al., 2013a,b), methane- and alkane-oxidizing archaea (Teske, 2024a,b), and hydrocarbon-oxidizing sulfate reducers (Teske, 2019). The distinct δ^13^C isotopic values of these carbon sources (Pearson et al., 2005) can be reflected in the biomass of these microbial populations. For example, strongly ^13^C-depleted filaments of sulfur-oxidizing *Beggiatoaceae* mats (average δ^13^C −42‰) indicate methane-derived carbon as a major carbon source (McKay et al., 2012), supported by the isotopic resemblance between *Beggiatoaceae* filaments and hydrothermal methane in Guaymas Basin (endmember δ^13^C value in hydrothermal sediments −42‰; Song et al., 2021). The high abundance of methane in hydrothermal sediments (multiple millimolar concentrations; McKay et al., 2016) suggests that this compound could percolate more widely through the trophic network of sedimentary bacteria and archaea; methane could compete with DIC and organic substrates as a major, generally available carbon source. Tracking the assimilation of methane and other fossil carbon sources into the bacterial and archaeal communities of surficial hydrothermal sediments (Pearson et al., 2005) motivated the current study.

Here we assess this possibility by determining the δ^13^C isotopic composition of bacteria and archaeal rRNA in the microbially densely populated sediments of Guaymas Basin, and by comparing them to δ^13^C isotopic signatures of potential carbon sources, such as DIC, methane and TOC that are abundant in Guaymas Basin and sustain microbial biomass. By comparing δ^13^C-rRNA signatures of natural bacterial and archaeal communities in hydrothermal and non-hydrothermal sediments, we focus on marker molecules with complex biosynthetic pathways that integrate the isotopic composition of cellular biomass (Nelson and Cox, 2004) and do not restrict the isotopic analysis to biomolecules with specific synthesis pathways and mutually exclusive source compounds; in other words, rRNA functions as an isotopic integrator of carbon flux into the microbial community (MacGregor et al., 2006). After it was initially suggested more than 20 years ago (Boschker and Middelburg, 2002), the ^13^C-rRNA isotope approach has been used to analyze bacterial communities and their preferred carbon substrates in sediments, using natural communities and ^13^C-labeled low-molecular-weight substrates in marine intertidal sediments (Miyatake et al., 2009, 2013), or salt marsh sediment communities amended with isotopically distinctive petroleum additions (Pearson et al., 2008). However, to the best of our knowledge, ^13^C-rRNA isotope studies have not targeted microbial communities in hydrothermal sediment samples that differ only by their *in-situ* spectrum of carbon sources, without any addition of labeled substrates.

As a general working hypothesis, we suggest that integrated hydrothermal carbon substrates are distinct from carbon sources in non-hydrothermal sediment, and these hydrothermal substrates influence the δ^13^C-rRNA composition for microbial (bacterial and archaeal) communities. Hydrothermal carbon sources would include hydrothermal methane (δ^13^C-CH_4_–43‰; McKay et al., 2016) and short-chain alkanes (ranging approx. From −13‰ for ethane toward −22‰ for hexane; Song et al., 2021). DIC would be locally variable between −25‰ and 0‰ (McKay et al., 2016), as illustrated by hydrothermal calcite (δ^13^C −9 to −14‰, Peter and Shanks, 1992). In methane-rich cores, methane-derived ^13^C-depleted carbon would enter the DIC pool, where it becomes available to autotrophs (McKay et al., 2016). Detrital organic matter of photosynthetic origin would be available everywhere in Guaymas Basin, regardless of local hydrothermal activity (ca. −20 to 25‰; Pearson et al., 2005).

Beyond our general working hypothesis that distinct carbon sources and their δ^13^C values would be reflected in microbial community rRNA as a whole, we also consider as a corollary the possibility of distinct δ^13^C values for bacterial and archaeal rRNA, since bacteria and archaea are members of distinct metabolic clades with different carbon substrate preferences and biochemical capabilities. For example, such differences are reflected in conspicuous δ^13^C differences for bacterial and archaeal intact polar lipids (Teske et al., 2002). Archaeal and bacterial candidates for metabolic groups with distinct isotopic signatures would include methane-oxidizing and DIC-assimilating ANME archaea (Biddle et al., 2012; Kellermann et al., 2012), aerobic methanotrophs (Teske et al., 2021), petroleum-oxidizing sulfate-reducing bacteria (Teske, 2019), DIC-assimilating sulfur-oxidizing and mat-forming *Beggiatoaceae* (MacGregor et al., 2013a), and DIC-assimilating nitrifying bacteria and archaea (Winkel et al., 2014). However, differences between bacterial and archaeal δ^13^C-rRNA values might be obscured by the compositional complexity of bacterial and archaeal communities in Guaymas Basin (Ramírez et al., 2021). Physiological ambiguity within specific microbial linages is another complicating factor, for example the ability of ANME-1 archaea to assimilate DIC (Kellermann et al., 2012), and the potential for acetate uptake in *Beggiatoaceae* (MacGregor et al., 2013b).

In addition to carbon sources, ^13^C-isotopic fractionation associated with particular carbon fixation pathways should play an important role in how source compounds are transformed into biomass and influence δ^13^C-rRNA values of bacterial and archaeal communities. The Wood-Ljungdahl pathway of autotrophic carbon fixation (integral for methanogens and methanotrophs) produces wide-ranging Δδ^13^C signatures but may reach maximal discrimination of up to 80‰, in contrast to weaker discrimination by the Calvin-Benson-Bassham cycle and the reverse TCA cycle (Δδ^13^C in the range of 20–30‰) (Garcia et al., 2021). In genomic surveys of Guaymas Basin microbial communities, the Wood-Ljungdahl pathway appeared most frequently, among the bacteria in Chloroflexi and Deltaproteobacteria, and among the archaea in members of the Desulfurococcales, Thermofilum, Methanomicrobia, and Altiarchaeales (Dombrowski et al., 2017). In contrast, the Calvin-Benson-Bassham cycle appeared only sporadically, in *Beggiatoaceae* and Parcubacteria; the reverse TCA cycle was identified in Thermoplasmatales and Archaeoglobales (Dombrowski et al., 2017). We caution that isotopic fractionations associated with autotrophic carbon fixation are likely to be diluted, since the vast majority of bacterial and archaeal lineages in Guaymas Basin sediments depend on heterotrophic fermentation and polymer degradation (Dombrowski et al., 2017, 2018).

Although the complexity of Guaymas Basin benthic microbial communities, and the diversity of uptake pathways and potential substrates, are likely to complicate ^13^C-based inferences on carbon sources, detecting any δ^13^C-rRNA signals that distinguish bacterial and archaeal communities at hydrothermal sediments from those at cold, inactive sediments would have diagnostic value. This study will demonstrate the inherent potential and limitations of this approach for microbial ecology at hydrothermal vents.

## Materials and methods

### Sediment sampling

Guaymas Basin sites were visited and sampled with R/V *Atlantis* and HOV *Alvin* during cruises AT15-40 and AT15-56 (Dec. 6–18, 2008; and Nov. 23-Dec. 4, 2009). *Alvin* dives targeted previously explored sampling areas in the southern Guaymas Basin with ample hydrothermal sediments and microbial mats (Teske et al., 2016). Thermal profiles were measured in surficial sediments using *Alvin’s* 50 cm heat flow probe.^1^ The 50 cm probe has thermal sensors every 10 cm, starting 5 cm under the attached plastic disk (the “puck”) that limits probe penetration and rests on the seafloor once the probe is inserted. After approx. 3–5 min, temperature readings stabilize and are recorded. After thermal profiling was concluded, push cores of approx. 12″ or 16″ (30–40 cm) length were collected, placed into the *Alvin* sampling basket, returned to the surface, and sub-sampled and frozen in the shipboard laboratory on the same evening. Depending on time of sampling, the time interval *ex-situ* before freezing was approximately 5–12 h. The time scale of hours is too short for rRNA growth shifts within a complex microbial community, as shown by ^18^O-tracking of rRNA and DNA synthesis in soil bacteria (Papp et al., 2018). Sampling site data for push cores used in this study are summarized in Table 1.

**Table 1:** Sampling locations of hydrothermal sediment cores used for rRNA extraction and δ^13^C-isotopic characterization.

Alvin dive and core number rRNA	Alvin dive and core number geochemistry	Sediment characteristics	Depth	Latitude/longitude	Metadata references
Background control					
4567-23	4567-28	Cold bare sediment	2,011 m	27N00.542/111W24.489	McKay et al. (2016), Lin et al. (2017)
Marker 14 site					
4569-5	4569-4	Temperate sediment	2,009 m	27N00.470/111W24.431	McKay et al. (2012, 2016), Lin et al. (2017), Song et al. (2021)
4569-16	4569-2	Hot white mat	2,009 m	27N00.470/111W24.431	McKay et al. (2012, 2016), Lin et al. (2017), Song et al. (2021)
4569-15	4569-9	Hot orange mat	2,009 m	27N00.470/111W24.431	McKay et al. (2012, 2016), Lin et al. (2017), Song et al. (2021)
Marker 27 site					
4572-15	4572-14	Temperate sediment	2,002 m	27N00.445/111W24.529	McKay et al. (2012)
4572-21	4572-20	Hot white mat	2,002 m	27N00.445/111W24.529	McKay et al. (2012)
4572-16	4572-18	Hot orange mat	2,002 m	27N00.445/111W24.529	McKay et al. (2012)
Marker 3 oily site					
4571-3	4571-4	Hot gray mat	2,007 m	27N00.466/111W24.438	McKay et al. (2016)
INSINC Mat					
4568-15	4568-13 & 14	Hot orange mat	2,001 m	27N00.443/111W24.543	Lin et al. (2017), Song et al. (2021)
Marker 14 Profiler mat					
4564-16	4564-14	Hot orange mat	2,008 m	27N00.466/111W24.425	Teske et al. (2016)
Megamat					
4490-8	4490-12	Hot oily mat	2,011 m	27N00.459/111W24.529	Song et al. (2021)
Marker 5					
4489-8	No geochemistry	Warm white mat	2,003 m	27N00.437/111W24.498	This study

Since sample size requirements precluded doing geochemistry and RNA extractions on a single core, cores were taken in pairs, one for geochemical analysis and one for RNA extraction.

### Geochemical methods

Porewater geochemistry was analyzed as described previously (Biddle et al., 2012). Sulfate concentration measurements were completed shipboard; after centrifuging sediment-filled 15 mL tubes, the overlying porewater was filtered through 0.45 μm filters, acidified with 50 μL of 50% HCl and bubbled with nitrogen for 4 min to remove sulfide. Sulfate concentrations were then measured shipboard using a 2010i Dionex Ion Chromatograph (Sunnyvale, CA, United States) through Ag^+^ exchange columns (Dionex) to remove Cl^−^ (Martens et al., 1999). For sulfide, 1 mL porewater samples were combined with 0.1 M zinc acetate and concentrations were analyzed spectrophotometrically on the ship (Cline, 1969). Headspace methane concentrations were determined onboard by standard gas chromatography with a flame ionization detector (FID), specifically using a HACH Carle Series 100 AGC Gas Chromatograph with an Alltech Molecular Sieve 5A packed column (80/100 mesh, 3.05 m length, 3.2 mm ID) and an 80 °C isothermal temperature profile. Stable isotopic compositions of these methane samples were measured post-cruise at UNC via gas chromatography-combustion-isotope ratio mass spectrometry (GC-C-IRMS) on a Finnigan MAT252 Isotope Ratio Mass Spectrometer, using a HP5890 Series II Gas Chromatograph with a HP Plot Q column (30 m length, 0.32 mm ID, 20 μm film thickness) and a 30 °C isothermal temperature profile. To measure DIC, 2 mL of unamended porewater from each sediment horizon were injected into evacuated serum vials (30 mL) and stored upside down at −20 °C. At UNC, the samples were thawed, and DIC was reacted to gaseous CO_2_ by adding 1 mL of a 30% phosphoric acid solution to each serum vial and shaking vigorously before GC analysis (Kelley et al., 1990). Stable isotopic values and concentrations of DIC were analyzed via coupled GC (Hewlett Packard, 5890) (Palo Alto, CA) and Isotope Ratio Mass Spectrometer (Finnigan MAT 252) (Hewlett Packard, Wilmington, DE, United States), as described previously (Rice et al., 2001). Porewater concentrations of dissolved organic acids were measured via HPLC (Albert and Martens, 1997). TOC and TN analyses (weight % and stable isotopes) were performed in the lab at UNC as follows: Solid sediment samples were lyophilized to remove any residual porewater. After lyophilization, the material was pulverized, weighed into combusted silver foil boats, and exposed to concentrated HCl vapor overnight in a closed vessel. Acid flushed samples were analyzed for total organic carbon (TOC) and total nitrogen (TN) content as well as carbon and nitrogen stable isotopic composition via flash combustion coupled with thermal conductivity detection (Carlo Erba NA 1500) and isotope ratio mass spectrometry (Finnigan MAT 252).

### rRNA isolation and electrophoresis

Total-community RNA was extracted from the frozen sediment using phenol-chloroform extraction with glass beads in an MSK-Zellhomogenisator (Braun Biotech International, Melsungen, Germany). RNA preparations from sediment samples were purified by isopropanol precipitation. The total RNA was quantified with a NanoDrop ND-1000 spectrophotometer (Thermo Scientific, Wilmington, DE). In order to estimate the amount of 16S rRNA in the total-community RNA, RNA extracts were visualized on a 5% polyacrylamide gel stained with ethidium bromide. The proportion of 16S rRNA among the RNA extracts was analyzed by comparing band intensities with known amounts of capture-isolated 16S rRNA by using ImageJ software.^2^

### Bead capture and separation of SSU rRNA

Small subunit rRNA was isolated from this mixed rRNA pool by magnetic bead capture using biotin-labeled domain-level probes (Bact338, Arch915) for the bacterial and archaeal domains (MacGregor et al., 2002). Magnetic-bead capture was performed following published protocols (Miyatake et al., 2009) using hydrophobic magnetic beads (Dynabeads MyOne Streptavidin T1; Invitrogen). The probes were hybridized overnight with total-community RNA extract at 20 °C on a rotator (Stuart SB3; Dynalab, Rochester, NY). The beads were washed, incubated with blocking agent and incubated with probe-target hybrid at room temperature on the rotator for 2 h. Beads with bound RNA were collected with the magnetic-particle concentrator, washed and the captured 16S rRNA was eluted in Milli-Q water (Millipore, Billerica, MA) at 90 °C for 3 min. Eluted 16S rRNA was precipitated with isopropanol and NaCl and finally dissolved in Milli-Q water. Between 300 and 600 ng C of captured 16S rRNA was pooled for isotope ratio measurement. Captured 16S rRNA was freeze-dried and one-by-one dissolved in freshly prepared, CO_2_-free Milli-Q water immediately before analysis by μEA-IRMS, to minimize the dissolution atmospheric CO2 into the sample. Protocol blanks with no RNA extract were also prepared with the same protocol.

### Methodological considerations concerning rRNA probes

Bacterial and archaeal 16S rRNA probes have inherent limitations. Some bacterial phyla (members of the heterotrophic Planktomycetales and Verrucomicrobia) require variants of probe 338 for detection (Daims et al., 1999). Thus, the range of probe 338 is properly described as “most bacteria” (MacGregor et al., 2006). The probe remains in use for quantitative studies, and it avoids the mutually similar bacterial/archaeal versions of “universal” probe 515 (Parada et al., 2016). Concerning archaeal probes, probe 915 is used very widely in analyses of marine sediments and subsurface sediments (Teske and Sørensen, 2008). Checking this primer against phylogenetically diverse reference sequences from marine sediments showed that existing mismatches (unavoidable in any primer) do not exclude any particular archaeal lineages (Teske and Sørensen, 2008). Even if domain-level probes fail to detect mismatched bacterial or archaeal lineages, any missed lineage would have to be sufficiently abundant and simultaneously have divergent d13C-rRNA signatures in order to impact the results.

### δ^13^C analysis of captured 16S rRNA

Isotope ratio analysis was performed by EA-IRMS consisting of a wet-oxidation interface (LC IsoLink; Thermo Fisher Scientific) coupled on-line to an isotope ratio mass spectrometer (Delta V Advantage; Thermo Fisher Scientific, Bremen, Germany) (Miyatake et al., 2009). Samples (50 μL containing 300–600 ng C of RNA) were directly injected into this μEA-IRMS operating in bulk injection mode. Standard curves were made with phthalic acid ranging from 0 to 1,000 ng of carbon. Stable carbon isotope ratios were expressed as δ^13^C values calibrated against the international standard Vienna Pee Dee Belemnite. The delta notation is defined as follows:

$\delta^{13}C_{\text{sample}}\;(‰)=[(R_{s}/R_{\mathrm{st}})\;-\;1]\times 1,000$

where *R_s_* is the ratio of ^13^C in the sample and *R_st_* is the ratio of the international standard VPDB (0.0111797). The measured δ^13^C values were corrected for the protocol blank as follows:

$\begin{array}{l} \;\delta^{13}C_{\mathrm{RNA}}\;(‰)\;=\;\\ \;[(\delta^{13}C_{\text{sample}}\times C_{\text{sample}})\;-(\delta^{13}C_{\text{blank}}\times C_{\text{blank}})]\\ /[\;C_{\text{sample}}-C_{\text{blank}}] \end{array}$

where  δ^13^C_sample_ is the δ^13^C value of the sample, C_sample_ is the amount of carbon in the sample,  δ^13^C_blank_ is the  δ^13^C value of the blank, and C_blank_ is the amount of carbon in the blank (Boschker, 2004). All samples were analyzed in duplicate.

## Results and discussion

### Site overview

**Figure 1:**
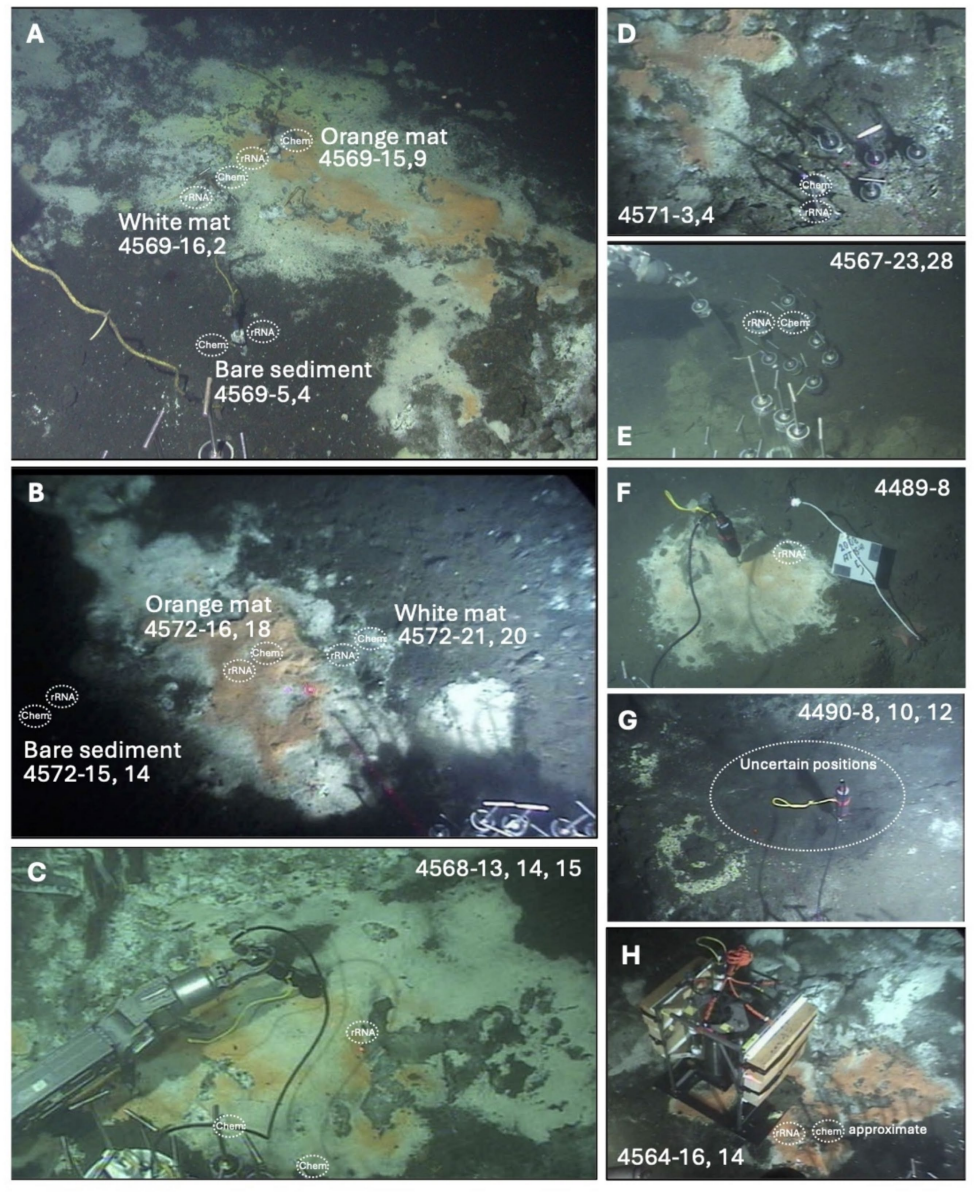
*In-situ* views of Guaymas Basin sampling sites sampled for this study. For each sampling site, the positions of sediment cores for geochemical and rRNA analysis are indicated, and annotated with *Alvin* core numbers. **(A)** Marker 14 hydrothermal area with white and orange *Beggiataoceae* mats, sampled during *Alvin* dive 4569. **(B)** Marker 27 hydrothermal area with white and orange *Beggiataoceae* mats, sampled during *Alvin* dive 4572. **(C)** INSINC hydrothermal area, dominated by white *Beggiatoaceae*, sampled during *Alvin* dive 4568. **(D)** Hot and oily sediments with sulfur precipitates, sampled during *Alvin* dive 4571. **(E)** Background sediments without microbial mats, sampled during *Alvin* dive 4567. **(F)** Moderately warm mat area at Marker 5, sampled for rRNA only during *Alvin* dive 4489. **(G)** Hot, alkane-rich Megamat area with surficial sulfur precipitates, sampled during *Alvin* dive 4490; frequent disturbance during repeated coring allows only an approximate localization of individual coring spots. **(H)** Approximate coring positions in orange mats near in-situ profiler, sampled during *Alvin* dive 4564 (Teske et al., 2016). *In-situ* photos were obtained from the Alvin frame grabber system (http://4dgeo.whoi.edu).

The sampling sites are located to either side of the approximate eastern boundary of the subsurface South Sill, which is hypothesized to focus hydrothermal flow along its edges (Teske et al., 2016). Except for the cold sediment control site, all sites were hydrothermally active, as evidenced by elevated temperatures, mats of white and orange filamentous *Beggiatoaceae* (MacGregor et al., 2013a, 2013b), or by sulfur precipitates on the sediment surface (Figure 1). The sites for which the most complete information was acquired are two hydrothermal hot spots with microbial mats (Markers 27 and 14), a hydrocarbon-rich site (Marker 3 oily site); and a background site with little or no detectable hydrothermal flow. Additional hydrothermal sites (INSINC Mat 1, Marker 5, Megamat, Mat profiler site) were analyzed in lesser detail but extended the sample set toward a greater variety of hydrothermal sampling sites (Figure 1; Table 1; Supplementary Figure S1).

### Caveats on core sampling

Although cores and temperature profiles were taken as near as possible to one another, the Guaymas sediments are quite heterogenous on small spatial scales. For example, one of the two “bare sediment” cores collected at Marker 27 (core 4752-15, Figure 1) had a chunky texture throughout on sectioning, most likely due to carbonate precipitation, while the other was smooth-textured. Methane-rich sediment cores can be disturbed by degassing as they are brought to the surface, resulting in turbid overlying water (e.g., Core 4572-18) or cracked and gapped sediments (e.g., Core 4572-16). Reported methane concentrations must therefore be considered underestimates, and downcore concentration profiles may not always be well preserved. Degassing is not expected to change stable carbon isotope ratios as such (Wallace et al., 2000), but depth profiles may be affected by mixing between sediment layers.

Repeated expeditions to Guaymas Basin have demonstrated flow changes on scales from minutes (McKay et al., 2016) to annual time scales (Teske et al., 2016), thus geochemistry, temperature, and microbial community structure should be regarded as temporary expressions of dynamically changing hydrothermal regimes.

### Geochemical profiles

**Figure 2:**
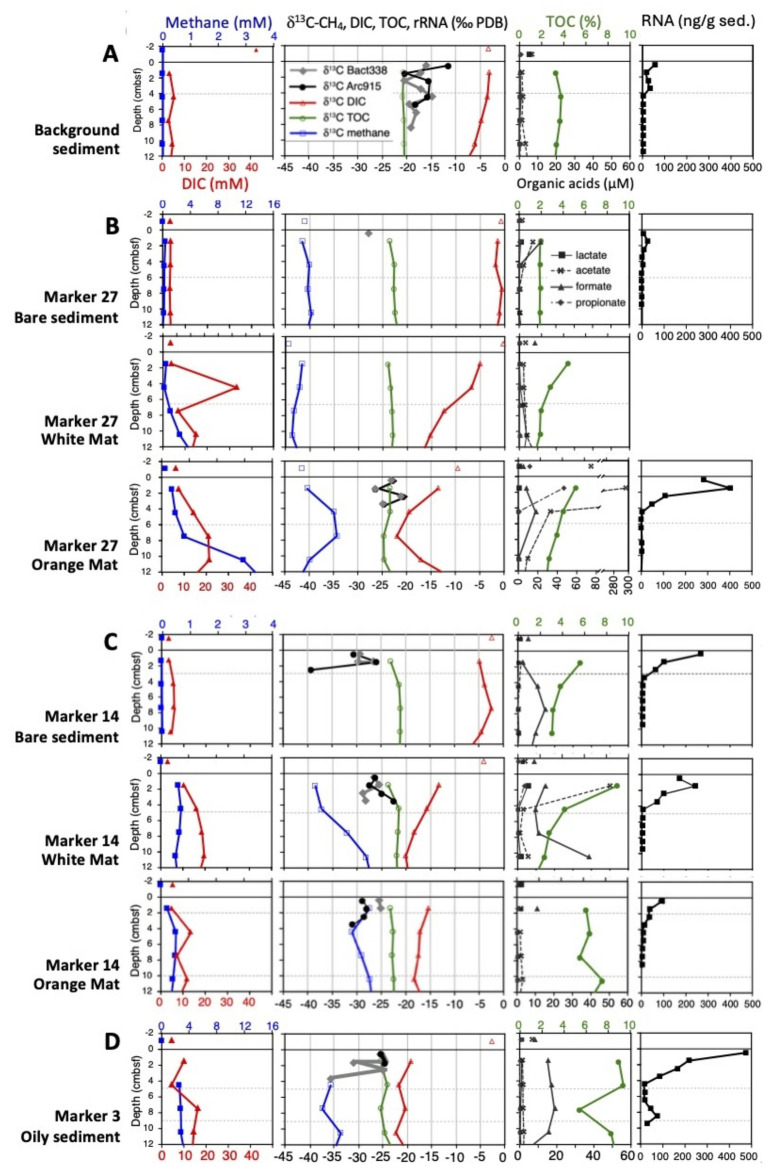
Biogeochemical profiles are shown for methane and DIC concentrations, δ^13^C values of methane, DIC, TOC and captured rRNA, TOC and organic acid concentrations, and total RNA concentrations, focused on the upper 12 cm sediment cores to resolve tightly clustered data points for δ^13^C-rRNA values and steep RNA concentration profiles in surficial sediments. Panel **(A)** shows the background sediment, panel **(B)** shows the mat gradient at Marker 27, panel **(C)** shows the mat gradient at Marker 27, and panel **(D)** shows the oily sediment at Marker 3. δ^13^C-rRNA rRNA values for the White Mat at Marker 27 are not plotted due to problems with the blank controls for these samples, but are available in the Supplementary text. Note the changing concentration scale for acetate at the Marker 27 orange core, and the different TOC scale at the oily site. A version of this figure showing more extensive biogeochemical and thermal profiles down to 40 cm depth is available as Supplementary Figure S1. All data are tabulated in Supplementary Table S1.

Geochemical data were collected to a depth of 20–40 cm for each site (Supplementary Table S1), in some cases including an overlying-water sample, and temperature profiles extended to 40 cm (Figure 2; Supplementary Figure S2). Stable carbon isotope ratios (δ^13^C_PDB_) were measured for methane, dissolved inorganic carbon (DIC), total organic carbon (TOC), and probe-captured SSU rRNA. Stable nitrogen isotope ratios (δ^15^N) were measured for TN. Total RNA was measurably recovered to approximately 5–10 cm depth.

We present the data by comparing the non-hydrothermal background site with hydrothermal gradients from hot spots with microbial mats that encompass three cores each, and then discussing different hydrothermal sites that are represented by single cores; some of these individual cores show unusual characteristics.

### Background sediment

Sediment temperatures at the background site (Figure 2A) were close to bottom water temperature (3 °C), warming by less than 1 °C with depth. Low sulfide concentrations below 0.01 mM and high sulfate concentrations between 24 and 26 mM indicated the absence of sulfide-rich, sulfate-depleted hydrothermal fluids. Methane concentrations were limited to the low micromolar range, but δ^13^C-CH_4_ values near −70‰ indicated microbial production (McKay et al., 2016). Dissolved inorganic carbon (DIC) concentrations ranged between 3 and 6 mM and are highest at the deepest depth sampled, 21–24 cmbsf; its δ^13^C values decreased from −3 toward −10.25‰ downcore, suggesting the impact of bioremineralization of organic matter. TOC concentrations remained near 3%, consistent with previous measurements for Guaymas sediments (de la Lanza-Espino and Soto, 1999). The stable carbon isotopic composition of TOC remained nearly constant between −20.3 and −20.9‰, within the range of −19 to −23‰ reported for photosynthetic production in Guaymas Basin (Druffel et al., 1992), and near the range for eukaryotic cholesterol in Guaymas Basin (δ^13^C −21 to −28‰, Teske et al., 2002); therefore we infer a predominantly detrital source of sedimentary biomass. Low-molecular weight organic acid concentrations (lactate, acetate, formate, proprionate) were always within or below the single digit micromolar ranges, indicating rapid microbial turnover (Supplementary Table S1).

TN concentrations were near 0.4% (weight percent sediment) in the upper sediments and decreased slightly downcore (Supplementary Table S1), similar to previously measured values for Guaymas background sediments (Ramírez et al., 2021). δ^15^N values remained consistently between 9.5‰ at the sediment surface and 10‰ downcore (Supplementary Table S1), very similar to non-hydrothermal sediments measured previously across the greater Guaymas Basin area (Ramírez et al., 2020). C/N ratios started at 9 at the surface and increased to 10.5 downcore (Supplementary Table S1) and thus showed moderate carbon enrichment compared to sediment cores collected far from hydrothermal areas in Guaymas Basin (Ramírez et al., 2020). Gradually decreasing TN concentrations and d^15^N values, and downcore increasing C: N ratios are consistent with microbial utilization of nitrogen compounds in sedimentary biomass (Ramírez et al., 2020).

### Hydrothermal gradients

In contrast to the background site, hydrothermal influence is evident in the geochemical characteristics of the Marker 27 and Marker 14 sites (Figures 2B,C). Here, white and orange sulfur-oxidizing *Beggiatoaceae* mats serve as visual proxies for hydrothermal gradients that intensify from bare sediment to white to orange mats (McKay et al., 2012). Detailed core-by-core descriptions are available in the Supplementary text; here we summarize general features of interest. Sulfide concentrations increase from micromolar in bare sediment to multiple millimolar concentrations underlying the mats (Supplementary Table S1), indicating that the filamentous, sulfide-oxidizing *Beggiatoaceae* respond to the availability of sulfide in hydrothermal sediments (McKay et al., 2012). Sulfate is never depleted in hydrothermal cores, indicating replenishment of this electron sink by hydrothermal circulation and seawater inmixing (McKay et al., 2016; Teske, 2024a, 2024b). The omnipresence of sulfate, the preferred electron acceptor for sulfate-reducing bacteria and archaea, likely limits the concentrations of low-molecular weight organic acids that serve as carbon sources for sulfate-reducing bacteria, and that remain in the low micromolar range (max. 50 μM acetate, 40 μM formate) in hydrothermal sediments. An acetate outlier near 300 μM is found just underneath orange mats at Marker 27. All cores from hydrothermal gradient sites show δ^13^C-TOC values that are slightly ^13^C-depleted (δ^13^C values of −21.3 to −23.6‰) compared to the background site, consistent with some incorporation of ^13^C-depleted carbon sources into biomass. Of the most abundant candidates for these light carbon sources (DIC and methane), methane is consistently ^13^C-depleted, with a δ^13^C-CH_4_ range of typically −30 to −40‰ (Supplementary Table S1). Relatively heavy δ^13^C-CH_4_ values in the range of −30 to −25‰, indicative of methane oxidation, occur in several hydrothermal cores and are linked to relatively ^13^C-depleted DIC values, indicating the preferential transfer of ^12^C methane carbon into the DIC pool by microbial methane oxidation (Supplementary Table S1).

Generally, the hydrothermal cores show a pattern of total nitrogen enrichment in surface samples, a likely consequence of nitrate uptake by mat-forming *Beggiatoaceae* (Buckley et al., 2019), followed by a rapid downcore decrease into the 0.1% TN range. Strong downcore increases in C/N ratios toward values around 20 and 30 are quite common in hydrothermal sediments, and extreme values above 100 are reached in a few cores (Supplementary Table S1). This conspicuous trend reveals the accumulation of nitrogen-depleted hydrocarbon compounds of hydrothermal origin that are diluting the nitrogen pool in hydrothermal sediments. Consistently, the δ^15^N values of TN in hydrothermal sediments remain a little below their counterparts from cold sediments (7–9‰, instead of 9.5–10‰) (Supplementary Table S1), suggesting reduced isotopic discrimination due to microbial utilization of increasingly scare nitrogen compounds.

Of the two hydrothermal gradient sites, the Marker 27 gradient (Figure 2B) was characterized by higher temperatures, by the presence of hydrothermal methane with δ^13^C values around −42‰ (McKay et al., 2016; Song et al., 2021), and by carbonate precipitates in the sediments, indicating a history of strong hydrothermal flow. Here, ^13^C-depleted δ^13^C-DIC values (−22‰) coincide with ^13^C-enriched δ^13^C-CH_4_ values (−35‰) between ca. 4 and 10 cm depth (Supplementary Table S1), indicative of methane-derived carbon entering the DIC pool. Compared to Marker 27, methane at the Marker 14 site is generally more ^13^C-enriched in downcore sediments; δ^13^C-CH_4_ values reach −28‰ in the orange mat core and −26‰ in the white mat core, whereas DIC reaches −18‰ and −23.5‰ (Supplementary Table S1), indicative of methane carbon entering the DIC pool. These trends are not limited to the upper 10 cm (as in Marker 27) but reach considerably deeper toward 15 and 30 cm depth, respectively, and suggest methane-derived carbon entering the DIC pool over a greater depth range (Supplementary Table S1). Thus, microbial methane oxidation (utilizing preferentially ^12^C-CH_4_) is more pervasive at the Marker 14 site compared to the Marker 27 site, most likely due to less extreme temperatures at Marker 14.

At both sites, DIC concentrations increased from mostly 3–4 mM in the bare sediments toward the 10–20 mM range in white and orange mat cores (Figures 2B,C). This DIC concentration increase was accompanied by increased ^13^C-depletion downcore that indicates the influx of a light carbon source into the DIC pool. This effect is most pronounced in the white mat core of Marker 14 where δ^13^C-DIC values reach −20 to −23‰ below 10 cm depth (Supplementary Table S1), and is consistent with concomitant microbial methane oxidation, and influx of methane-derived ^13^C-depleted carbon into the DIC pool, in the same core. It would be interesting to have bacterial and archaeal δ^13^C-rRNA data for these extended depth intervals (not just the top 4 cm; Supplementary Table S1), and to observe the impact of methane oxidation on bacterial and archaeal rRNA.

Two hydrothermal sites with *Beggiatoaceae* mats where single cores were analyzed (INSINC site and Microprofiler site, Table 1) share the general characteristics of organic matter remineralization to DIC, high methane concentrations, and isotopic evidence for methane oxidation (Supplementary text; Supplementary Figure S2). The Microprofiler site also shows hydrothermal nitrogen depletion that characterizes the Marker 27 and Marker 14 gradient sites (Supplementary Table S1).

### Unusual hydrothermal sites

A few meters distant from Marker 14, hydrothermal core 4571-4 (Figure 2D) was collected from oil-rich sediment at the moderately hot Marker 3 site (63.2 °C at 40 cmbsf). Abundant reddish-brown oil bubbles in the freshly recovered core, and a strong petroleum smell indicated hydrothermal petroleum seepage at this location. Young petroleum is generated under high heat and pressure within the upper sediment column of Guaymas Basin (Peter et al., 1991). This hydrocarbon-rich core yielded the highest TOC abundances (reaching 12–15 mM) and the most N-depleted C/N ratios (>60) of the entire sample set. Consistently high methane concentrations combined with increasingly heavy (^13^C-enriched) δ^13^C-CH_4_ values that reach −16‰ at 40 cm depth indicate the importance of methane oxidation in this core; these are the most strongly ^13^C-enriched δ^13^C-CH_4_ values found in the entire dataset. As the methane pool becomes isotopically heavier, the DIC pool becomes lighter. DIC concentrations increasing downcore to >20 mM coincide with increasingly light δ^13^C-DIC values that start at −19.2‰ at the surface and reach near −25‰ downcore. These are the most δ^13^C-depleted DIC values in the entire dataset. Influx of ^13^C-depleted methane-derived carbon into the DIC pool would explain these observations; organic matter could not be the sole source since it is not sufficiently ^13^C-depleted (δ^13^C-TOC between −19 and −23‰; Druffel et al., 1992). At present we do not have δ^13^C data for hydrothermal petroleum in this core, but previous δ^13^C measurements of bulk petroleum in Guaymas Basin yielded values in range of −20.5 to −23‰, with aliphatic hydrocarbons between −21.7 and −22.9‰ and polyaromatic asphaltenes between −20.5 and −20.8‰ (Schoell et al., 1990). Thus, petroleum compounds reflect the ^13^C content of detrital organic matter, and would be insufficiently ^13^C-depleted to account for the strongly ^13^C-depleted δ^13^C-DIC pool by microbial oxidation of petroleum compounds; a methane-derived contribution to the DIC pool is therefore essential.

The hydrothermal Megamat site (core 4490-10) stands out in several ways. Among all hydrothermal sites, this one has the weakest isotopic evidence for anaerobic methane oxidation since its δ^13^C-CH_4_ values remain consistently near −42‰ (Supplementary Figure S2); this isotopic signature represents the hydrothermal background that characterizes hot fluids before the impact of microbial oxidation (Song et al., 2021). The abundant DIC pool (>10 mM) remains isotopically heavy (δ^13^C -DIC is changing from −3 to −6‰ downcore) and is barely impacted by ^13^C-depleted methane carbon (in contrast to other hydrothermal sites, see Supplementary Figure S2). We suggest that a TOC-derived contribution is slowly impacting δ^13^C -DIC values downcore, analogous to the situation in background sediment. Also unusual at this site are its high concentrations of light alkanes that increase with chain length, from near 0.1 millimolar ethane to 1.5 millimolar pentane (Song et al., 2021). The δ^13^C values for light alkanes at Megamat change from −13 to −14‰ for ethane toward −19‰ for propane, and −20 to −21‰ for butane and pentane; a similar trend of increasing ^13^C depletion with alkane chain length is also seen at other sites (Supplementary Table S1). This trend illustrates the origin of ethane from DIC carbon and TOC-derived acetate carbon; subsequent condensation reactions that add TOC- and acetate-derived carbon shift the δ^13^C values of propane, butane and pentane toward δ^13^C-TOC, near −21‰ (Song et al., 2021). These short-chain alkanes constitute a microbial carbon source that is exploited by specialized alkane-oxidizing microbial consortia in Guaymas Basin (Laso-Pérez et al., 2016; Hahn et al., 2020), and alkane bioremineralization might impact the δ^13^C-DIC values at this site.

When comparable alkane data exist for other sites (Marker 14 and INSINC mat), short-chain alkane concentrations remain always in the low micromolar range and reach at most 50 μm for ethane (Supplementary Table S1). Yet regardless of low abundances, δ^13^C profiles for short-chain alkanes show the isotopic imprint of microbial oxidation where the residual alkane pool becomes ^13^C-enriched. For core 4569-3, δ^13^C -ethane values converge toward 0‰ locally (Supplementary Table S1). Similar results were seen for alkane-containing sediment cores in other Guaymas Basin studies (Dowell et al., 2016; Song et al., 2021; Teske, 2024a, 2024b).

### RNA concentrations and stable carbon isotopes of SSU rRNA

While the downcore extent of geochemical profiles was usually limited only by sediment recovery and extended in long cores toward 40 cm depth, the profiles of RNA concentration and δ^13^C rRNA were limited to surficial sediments where microbial biomass is concentrated, as shown by direct cell counts (Meyer et al., 2013), lipid concentration profiles (Schouten et al., 2003), and dilution culture to extinction (Teske et al., 2009). Sediment depth impacts not only general microbial population density, but also the relative proportions of bacteria and archaea, and thus, their contributions to the sedimentary rRNA pool. In high-throughput amplicon sequencing studies relying on “universal” (bacterial/archaeal) 16S rRNA primers, ASV frequencies show an archaeal contribution of ca. 10–20% (range ca. 3–30%) for surficial hydrothermal sediments and ca. 5% for cold background sediment; the archaeal contribution increases with sediment depth and temperature (Ramírez et al., 2021). A similar trend was found in a qPCR analysis that compared bacteria and archaea in mat-covered seep sediments of the Sonora Margin, using different primers than this study (Vigneron et al., 2013). The archaeal contribution in surficial sediments ranged—with considerable site variability—from less than 10% to ca. 25% in surficial sediments (0–4 cm), but increased in deeper sediments and dominated below 6–12 cm depth, again depending on site. Thus, surficial sediments (analyzed in this study) likely yielded more bacterial than archaeal rRNA. The rapidly declining overall rRNA content in downcore sediments precluded the recovery of archaeal-enriched rRNA from deeper sediment layers.

The dense microbial populations in surficial sediments act as an active filter that assimilates and processes carbon sources that migrate into surficial sediments following hydrothermal circulation; the ^13^C-isotopic composition of bacterial and archaeal rRNA is recording the impact of these combined carbon sources. In the following sections that compare the δ^13^C values of rRNA and the δ^13^C values of different microbial carbon sources, the implicit assumption is made that carbon assimilation into rRNA does not cause major isotopic fractionation in itself, as shown in control experiments with *E. coli* and various organic carbon sources (MacGregor et al., 2002). Thus, the δ^13^C values of microbial rRNA can be used as a diagnostic tool to infer microbial carbon sources to some extent.

Total RNA concentrations (ng/g sediment) within hydrothermal sediment cores ranged over four orders of magnitude within short downcore distances, with one order of magnitude decrease for 2 or 3 cm sediment depth: from approx. 100 to 400 ng/g in surficial sediments at 0.5 and 1.5 cm depth, toward 1 to 4 ng/g at 4–5 cm depth, and 0.01 to 0.04 ng/g at 10 cm depth and below (Supplementary Table S1). Cold Background sediments showed slightly lower RNA concentrations that started at 50 ng/g in surficial sediments. The bare sediment core at Marker 27 contained the lowest RNA concentrations in surficial sediments, 27 ng/g (Supplementary Table S1). In most hydrothermal cores, δ^13^C values for bacterial and archaea rRNA could be determined only for surficial sediments down to 3–4 cm depth. Interestingly, sensitivity was higher for the cold background sediments where δ^13^C measurements for bacterial and archaea rRNA could be obtained down to 8–9 and 5–6 cm depth, respectively (Supplementary Table S1); the difference in sensitivity suggests that hydrocarbon content in hydrothermal sediments interferes with RNA recovery (Ramírez et al., 2024) and subsequent δ^13^C-rRNA analyses.

Results for δ^13^C analyses of probe-captured bacterial and archaeal rRNA showed consistent differences between the background site and the hydrothermal sites. The cold background sediments (core 4567-23) yielded bacterial and archaeal rRNA with δ^13^C values between −15 and −20‰, except for an archaeal rRNA outlier with a δ^13^C value of −11.35‰ in the 0–1 cm sample. The dominant carbon source at the cold background site is sedimentary TOC of photosynthetic origin (δ^13^C near −20.5‰), possibly supplemented by some DIC assimilation as a secondary source (δ^13^C-DIC changing from −3 to −10‰ downcore, reflecting the increasing imprint of bioremineralization). The ^13^C-replete DIC contribution appears to be strongest in the archaeal rRNA in surficial sediment, where autotrophic Thaumarchaeota are present (Teske et al., 2021); these archaea assimilate DIC using the 3-Hydroxyproprionate/4-hydroxybutyrate cycle with minimal fractionation (Δδ^13^C-DIC 0–4‰) (Garcia et al., 2021). Hydrothermal cores yielded bacterial and archaeal rRNA that was considerably more ^13^C-depleted, in most cases between −25 and −30‰ (Supplementary Table S1). Isotopic differences or trends that consistently distinguish bacterial from archaeal rRNA across the dataset were not found. Strongly ^13^C-depleted outliers for bacterial and archaeal rRNA values were observed at 3–4 cm depth in the hydrocarbon core 4571-3 (δ^13^C-rRNA_bact_ = −35‰) and at 2–3 cm depth in Marker 14 core 4569-5 (δ^13^C-rRNA_arch_ = −39.12‰).

### The impact of methane on rRNA ^13^C-isotopic composition

**Figure 3:**
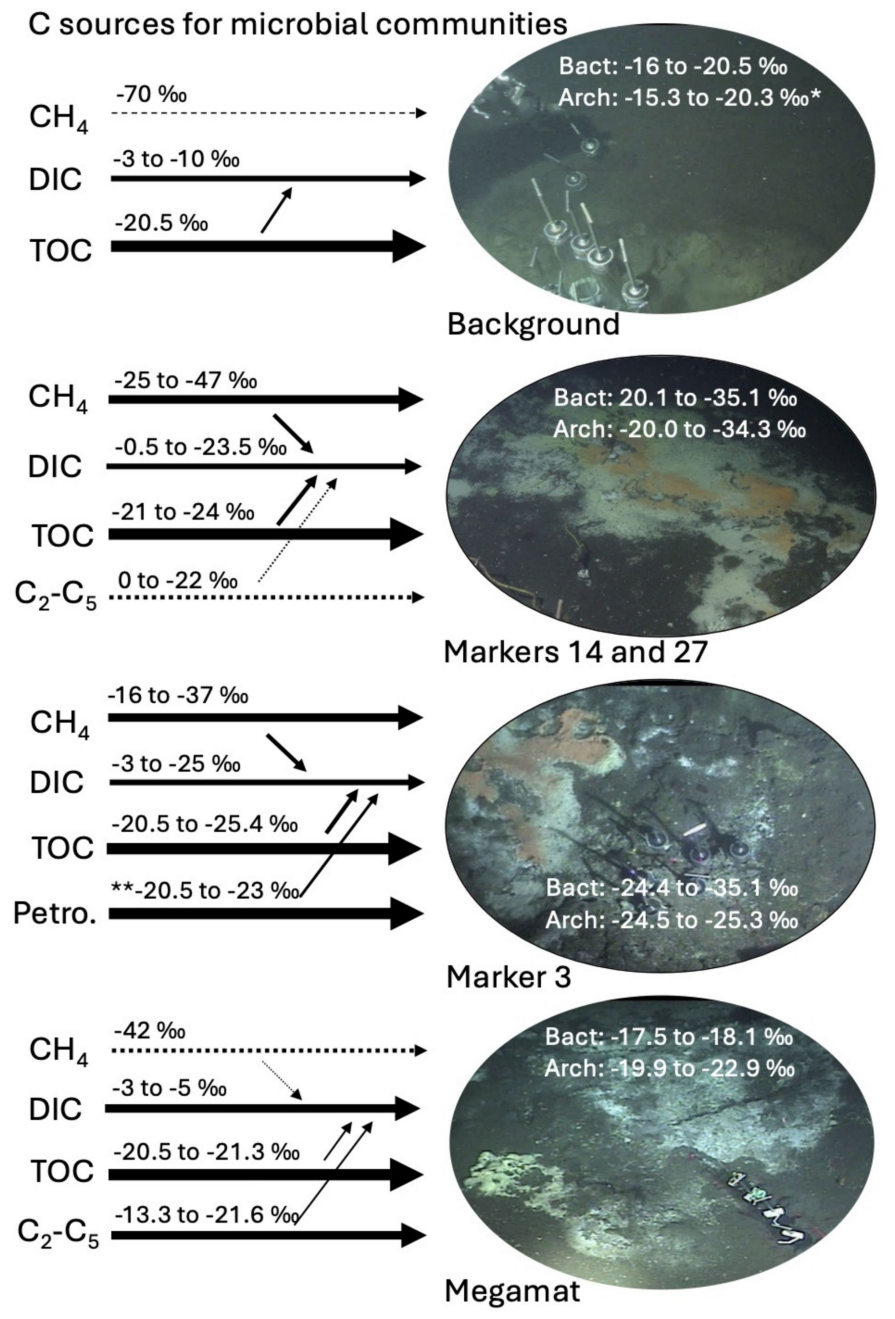
Synthesis sketch for microbial carbon sources in different hydrothermal and background sites, including methane, DIC, sedimentary organic matter (TOC), light alkanes and petroleum. Methane and other hydrocarbons. Arrow thickness represents the inferred relative contributions of carbon sources to microbial biomass. Smaller arrows converging on DIC indicate microbial oxidation of TOC, methane, and fossil carbon sources. These processes are marked by simultaneous ^13^C increase of the source compounds and ^13^C decrease of the DIC pool (Supplementary Table S1), as ^12^C-carbon compounds are preferentially utilized and remineralized by microbes. *, excluding outlier of −11.35‰ for archaeal δ^13^C-rRNA in surficial cm layer of background core 4567-28. **, literature δ^13^C value for Guaymas Basin petroleum (Schoell et al., 1990).

Which geochemical factors could be linked to the difference in δ^13^C-rRNA values between background sediment and hydrothermal sediment? Hydrothermal sediments contain methane, porewater DIC and sedimentary TOC that are consistently more ^13^C-depleted than their counterparts in background sediment (Figure 3). Among these three carbon pools, methane is comparatively more δ^13^C-depleted than DIC and TOC, except in specific cores and samples where microbial methane oxidation is highly active and transfers methane-derived light carbon into the DIC pool. In first approximation, we suggest that δ^13^C-depleted methane-derived carbon is incorporated into microbial biomass alongside DIC and TOC (Figure 3), potentially through methane-oxidizing ANME-1 archaea that are ubiquitous in Guaymas Basin hydrothermal sediments (Dowell et al., 2016; McKay et al., 2016) and remain active across the mesophilic and thermophilic temperature spectrum (Holler et al., 2011; Wegener et al., 2015; Benito Merino et al., 2022). However, since ANME-1 archaea appear to assimilate DIC to a greater extent than methane (Treude et al., 2007; Kellermann et al., 2012), ANME-2 archaea and aerobic methanotrophic bacteria (Teske et al., 2021) need to be considered as major “methane assimilation portals” in surficial sediments. ANME-1 archaea could assimilate methane-derived carbon after oxidization to DIC, consistent with the unusually wide range of ^13^C-fractionation observed for ANME-1 cells and consortia (Orphan et al., 2002). In hydrothermal methane-rich sediments, methane-derived carbon contributions increase δ^13^C-depletion of bacterial and archaeal rRNA beyond what is observed in cores where carbon sources are limited to DIC and TOC. In other words, without a contribution of methane-derived carbon to the DIC pool, utilization and remineralization of photosynthetic biomass would not produce sufficiently light ^13^C-DIC values. The observation that bacterial and archaeal δ^13^C-rRNA values are not systematically different from each other suggests that methane-derived carbon has permeated the microbial trophic network, independently of specific “gateway” bacteria and archaea and their uptake mechanisms. Yet, the diversity of autotrophic and heterotrophic carbon assimilation pathways that have been found in genomic surveys of Guaymas Basin (Dombrowski et al., 2017, 2018) suggests that different lineages make distinct but presently unresolved contributions to the resulting domain-level δ^13^C-rRNA signatures.

### Other carbon sources impacting rRNA ^13^C-isotopic composition

When methane is not the dominant microbial carbon source, the imprint of methane-derived carbon in the bacterial and archaeal rRNA pools decreases in importance compared to other carbon sources. This appears to be the case in hydrothermal core 4490-8 from the alkane-rich Megamat site, where δ^13^C-rRNA values are distinctly heavier than the −25 to −30‰ range that is typical for rRNA from all other hydrothermal sediments, and instead resemble the δ^13^C-rRNA values of background sediment (Figure 3). Interestingly, Megamat is also the only site with a clear isotopic difference between bacterial and archaeal rRNA (δ^13^C-rRNA_bact_ −17.5 to −18.1‰; δ^13^C-rRNA_Arch_ −19.9 to −22.9‰) (Figure 3). Local methane is abundant (2–3 mM), but remains highly ^13^C-depleted throughout the core (δ^13^C-CH_4_ near −42‰ abiotic hydrothermal baseline), indicating limited methane oxidation and limited influence on the relatively heavy δ^13^C-rRNA values (which resemble those of the background sediment where methane is barely detectable). Two factors might explain the reduced ^13^C depletion in microbial rRNA at this site. Autotrophic assimilation of abundant DIC (8–16 mM) with low degrees of ^13^C-depletion (δ^13^C-DIC approx. −3 to −6‰) could impact the δ^13^C-rRNA values. In this scenario, the assimilation of moderately ^13^C-depleted DIC (possibly by omnipresent Chloroflexi and Bathyarchaeota using the Wood-Ljungdahl pathway; Dombrowski et al., 2017, 2018) would contribute to reduced ^13^C-depletion for rRNA in the Megamat core and in the background core. The second possibility is that locally dominant short-chain alkanes with relatively heavy δ^13^C values ranging from −13.3 to −21.6‰ (Figure 3) are microbially assimilated by specialized alkane-oxidizing bacteria and archaea, and this causes the unusually heavy ^13^C-rRNA biosignatures.

Since the elevated short-chain alkane content distinguishes the Megamat core from all others, we speculate that this unusual substrate spectrum favors distinct alkane-processing pathways and key enzymes among bacteria and archaea, and that these might be responsible for the isotopic differences between bacterial and archaeal rRNA that appear specifically in this core. Bacteria would oxidize and assimilate alkanes and aromatics via fumarate addition (Kniemeyer et al., 2007), whereas archaeal alkane oxidation and assimilation would proceed via alkyl-coenzyme M reductase-dependent activation (Laso-Pérez et al., 2016; Zehnle et al., 2023). For archaeal methyl coenzyme M reductase, breaking the C-H bond of free methane before formation of the C-S bond in the activated methyl-coenzyme M complex has been identified as the rate-limiting step that governs kinetic isotope effects (Scheller et al., 2013). For bacterial fumarate addition, the cleavage of a C-H bond during fumarate addition to a hydrocarbon substrate is the isotopically sensitive step (Herrmann et al., 2009). It would be fascinating to observe the isotopic consequences of bacterial fumarate addition and archaeal methyl/alkyl coenzyme M activation running next to each other in controlled enrichment experiments.

## Conclusion

Sedimentary carbon pools—detrital organic matter, DIC, methane, alkanes, petroleum—contribute differently to bacterial and archaeal δ^13^C-rRNA values, depending on substrate availability (Figure 3). The isotopic imprint of ^13^C-depleted methane is conspicuous in bacterial and archaeal δ^13^C-rRNA values, and is understood as consequence of its abundance and ubiquity in hydrothermal sediments of Guaymas Basin. When methane is not available, the δ^13^C-rRNA values for bacteria and archaea become less ^13^C-depleted and indicate the preferential utilization of detrital organic matter (Figure 3). When δ^13^C-depleted methane competes with high concentrations of δ^13^C-replete short-chain alkanes, δ^13^C-rRNA values for bacteria and archaea are heavier than those in methane-rich, alkane-poor sediments, suggesting the possibility of alkane assimilation by bacteria- and archaea-specific pathways (Figure 3). To summarize, we note that the availability of different sedimentary or hydrothermal carbon sources is reflected in changing δ^13^C-rRNA values for bacterial and archaea, with the presence of hydrothermal methane as the most noticeable signal but other factors contributing as well.

## Data Availability

The original contributions presented in the study are included in the article/Supplementary material, further inquiries can be” directed to the corresponding author.

## Footnotes

^1^ https://ndsf.whoi.edu/alvin/using-alvin/sampling-equipment/

^2^ http://rsb.info.nih.gov/ij/
