# Supplementary material for: Tracing microbial carbon sources in hydrothermal sediments by 13C isotopic analysis of bacterial and archaeal ribosomal RNA
Source: Front Microbiol. 2025 Oct 16;16:1680337. doi: 10.3389/fmicb.2025.1680337 (PMC12574382; doi:10.3389/fmicb.2025.1680337)
Supplement: Supplementary file 2 [file Data_Sheet_1.docx]

**Supplementary online Materials**

**Tracing microbial carbon sources in hydrothermal sediments**

**by ^13^C isotope signatures of bacterial and archaeal ribosomal RNA**

Barbara MacGregor, Henricus T.S. Boschker, Dan Hoer, Daniel B. Albert, Howard Mendlovitz, Andreas Teske

**Description of hydrothermal gradient sample sets**

**Marker 14 gradient***.* At present, this is the best-studied three-core transect of a *Beggiatoa*-covered hydrothermal hot spot in Guaymas Basin, complete with geochemistry (McKay et al. 2012), 16S rRNA gene sequences (McKay et al. 2016), metagenome-derived genomes (Dombrowski et al. 2018), and short-chain alkane concentrations and δ^13^C values (Song et al. 2021). In the center of the hot spot (core 4569-9), temperatures increase from 13°C at the surface of the mat to 94°C at 40 cmbsf, and geochemical data indicate anaerobic methane oxidation at high temperatures. Methane concentrations are lowest at the surface (0.95 mM) and remain between 1.5 mM and 2.3 mM throughout the rest of the core. Methane is ^13^C-enriched at the surface (-27.41 ‰) and becomes lighter downcore, reaching -37.69 ‰ at the last depth sampled where the interpolated temperature from *in situ* measurements was 83.6°C. DIC concentrations fluctuate considerably between 5 mM and 25 mM and δ^13^C-DIC values are fluctuating between -12 ‰ and -18 ‰, indicating that DIC derived from methane oxidation and organic matter remineralization are mixing with Guaymas Basin hydrothermal fluids (δ^13^C-DIC -9.4‰) and bottom water (-0.6‰) (Pearson et al., 2005). Sulfide concentrations at the top and bottom of the core are approximately 0.9 mM, with a peak concentration of 1.9 mM at 9-12 cmbsf. No significant sulfate depletion is observed and sulfate concentrations vary within 1.4 mM throughout the core, indicating strong hydrothermal circulation and sulfate replenishment below the sediment surface.

These indicators for a high-temperature methane-oxidizing sediment characterize, in attenuated form and under a milder temperature gradient (from 3°C at the surface to 63.2°C at 40 cmbsf), the second step in this transect where sediment covered by white *Beggiatoa* is surrounding the central orange region (geochemistry core 4569-2). Methane concentrations range from 2.1 mM to 2.9 mM throughout the core and methane becomes most ^13^C-enriched (-25.52 ‰) midcore at a depth of 21-24 cmbsf and temperature of 41.5°C. Downcore increasing DIC concentrations and gradually more negative δ^13^C-DIC values suggest the influence of bioremineralization and methane oxidation; these trends attenuate and reverse themselves after approx. 20 cm depth. The sulfate concentration profile is highly variable but does not reach sulfate depletion anywhere, although sulfide accumulation downcore indicates active sulfate reduction.

Beyond the edge of the orange and white *Beggiatoa* mat (geochemistry core 4569-4), bare sediments are still warm; temperature increase from 3.3°C at the sediment-water interface to 23.1°C at a depth of 40 cmbsf. Methane accumulates with depth towards approx. 1 mM, while changing from -47.58 ‰ δ^13^C-CH_4_ at the surface to ca. -28 ‰ at a depth of 21 – 24 cmbsf with a corresponding temperature of 14.4°C, indicating low-temperature methane oxidation. The DIC concentration remains variable but generally increases from 4.1 mM at the surface to a maximum of 9.3 mM at a depth of 24 – 33 cmbsf where temperatures range between 15°C and 19°C. DIC is ^13^C-enriched between the surface and 9 cm, and then becomes ^13^C -depleted towards -12.13 ‰ at 30 cm, reflecting the influence of different biogenic ^13^C -depleted DIC pools. Sulfate remains abundant in the 25 mM range throughout the core, whereas sulfide accumulating to 1 mM downcore indicates active sulfate reduction.

**Marker 27 gradient**. At this gradient, metadata are limited to the thermal gradient and porewater geochemistry (McKay et al. 2012), yet the overall picture of geochemical gradients indicating methane oxidation, organic matter remineralization to DIC, and sulfate reduction is similar to the sediments at Marker 14. In hot sediments under the orange mat (core 4572-18, 101°C at 45 cmbsf), very high methane concentrations of 15 mM near 14 cm depth decrease towards the sediment surface and coincide with heavier δ^13^C-CH_4_ values (from -42.2‰ to -34.9‰), indicative of methane oxidation. A distinct DIC concentration peak of 21 mM at 7 to 10 cm depth coincides with the local δ^13^C-DIC minimum of -22‰, consistent with DIC contributions from the bioremineralization of photosynthetic biomass and methane oxidation. In the white mat core (4572-20), the temperatures below 30 cm exceed those of in the orange mat core (101°C at 45 cmbsf) whereas the geochemical gradients occur in attenuated form. Methane concentrations reach 10 mM at 25 cm depth, and isotopic evidence for methane oxidation is limited to a very small change in δ^13^C-CH_4_ values (-43.6 to -41.6 ‰) within the upper 10 cm of the sediment. The DIC concentration peak (14.8 mM at 10 cmbsf) and the nearby local δ^13^C-DIC minimum (-17.1‰ at 13 cmbsf) are attenuated compared to the orange mat core. The sulfate concentration minima and matching sulfide maxima are shifting downcore, from ca. 8 cm depth underneath the orange mat to ca. 14 cm below the white mat.

The bare sediment at Marker 27 resembled the periphery core of Marker 14 in temperature (approx. 20°C at 40 cmbsf), and showed some parallels with the cold background site: all measured profiles were relatively constant with depth; sulfide and methane do not accumulate within the cored sediment depth, and DIC concentrations remain consistently low, between 2.9 and 3.7 mM. Interestingly, δ^13^C-DIC values remained near -3 to -1‰ throughout the core and were thus more positive than δ^13^C-DIC values in the background core that showed the influence of biomineralization. However, residual hydrothermal methane remained detectable, and methane concentrations at the peripheral site (0.1-0.4 mM) were high enough for isotope determinations, showing that δ^13^C-CH_4_ increased from -43.4‰ (the hydrothermal methane signature, McKay et al. 2016) at 30-33 cm depth to -39.7‰ at 9-12 cm. TOC overall is slightly depleted compared to the background site (δ^13^C of -21.3‰ to -23.6‰). Thus, both bare sediment cores at Markers 14 and 27 retain some hydrothermal influence and show evidence of microbial methane cycling, in methane oxidation and incorporation into biomass.

**Microprofiler** **site**. This orange mat site (geochemistry core 4564-14) shares the characteristics of other orange mat sampling sites: high temperature (95°C at 40 cmbsf), δ^13^C-CH_4_ values indicating ^13^C-CH_4_ enrichment and methane oxidation in the upper half of the core, and δ^13^C-DIC values of -13 to -18‰ that indicate a mixture of hydrothermal and biogenic DIC. Sulfate concentrations decrease to ca. 15 mM at 20 cmsbf, and sulfide occurs in 1-2 mM concentrations throughout the core, indicating microbial sulfate reduction and thus bioremineralization. These profiles were determined by shipboard core slicing and porewater analysis in 3 cm intervals (as all other profiles in this study), but nearby *in-situ* microprofiler measurements (Winkel et al. 2014, Teske et al. 2016) show the same profiles in millimeter-scale resolution. The microprofiler results highlight important details that should apply fundamentally to all mat-covered sediments, for example the immediate appearance and accumulation of sulfide below the sediment surface, and the instantaneous consumption of oxygen at the sediment/water interface.

**INSINC site**. This orange and white mat site (geochemistry cores 4568-13 and 14) resembles other hot sites by its high temperature (115°C at 45 and 96°C at 40 cmbsf, respectively), and shares their geochemical signatures of microbial methane cycling and sulfate reduction. High methane concentrations in the 2-3 mM range and localized δ^13^C-CH_4_ maxima (-35 % and -16%, respectively) indicate ^13^CH_4_ enrichment and methane oxidation in the upper sediment layers, and variable δ^13^C-DIC values ranging from of -8 to -19.5‰ indicate mixtures of hydrothermal and biogenic DIC. Sulfate concentrations remain between 22 to 26 mM in both cores, and sulfide occurs in both cores with maxima of 0.8 and 1.6 mM, respectively, indicating microbial sulfate reduction and thus bioremineralization. Sediment cores from this site were used for a detailed study of organic matter mobilization under hydrothermal conditions (Lin et al. 2017), and for enrichments of thermophilic, methane-oxidizing ANME-1 archaea (Holler et al., 2011) and for light alkane-oxidizing *Ca*. Syntrophoarchaeum (Laso-Pérez et al., 2016).

**Microscopic cell counts**. Bacterial cells were fixed with formalin (2% final conc.) and stained with 4′, 6-diamidino-2-phenylindole on a 0.2-mm polycarbonate filter (DAPI; Porter and Feig, 1980). Cells were counted in a Whipple grid field (71.5 x 71.5 μm) at 1250 x magnification on an Olympus epifluorescence microscope. 10 fields per slide were counted. DAPI cell counts for Guaymas Basin sediments were consistently at ~10 x 10^10^ cells cm^-3^ for background and hydrothermal cores (Figure S3). In contrast to exponentially declining RNA yields downcore, cell counts remained high, within 1 to 4 x 10^10^ cells cm^-3^, also in deep and hot sediment samples.

**Samples with background problems**. The blank controls for Marker 27 white Mat showed a twenty-fold elevated carbon load (near 3500 ng/50 µl sample) compared to the rRNA samples (100-250 ng/50 µl samples), suggesting that the results should be taken with caution. Regardless of the problematic controls, the bacterial and archaeal δ^13^C-16S rRNA data were in themselves not unusual (-26.8/-31.9 ‰ and -27.6/-29.4 ‰ for bacterial rRNA in samples 1 and 2, respectively, -32.1 and -34.2 ‰ for archaeal rRNA in samples 1 and 2). Exact centimeter depths for these samples are no longer available due to documentation and curation gaps.

**
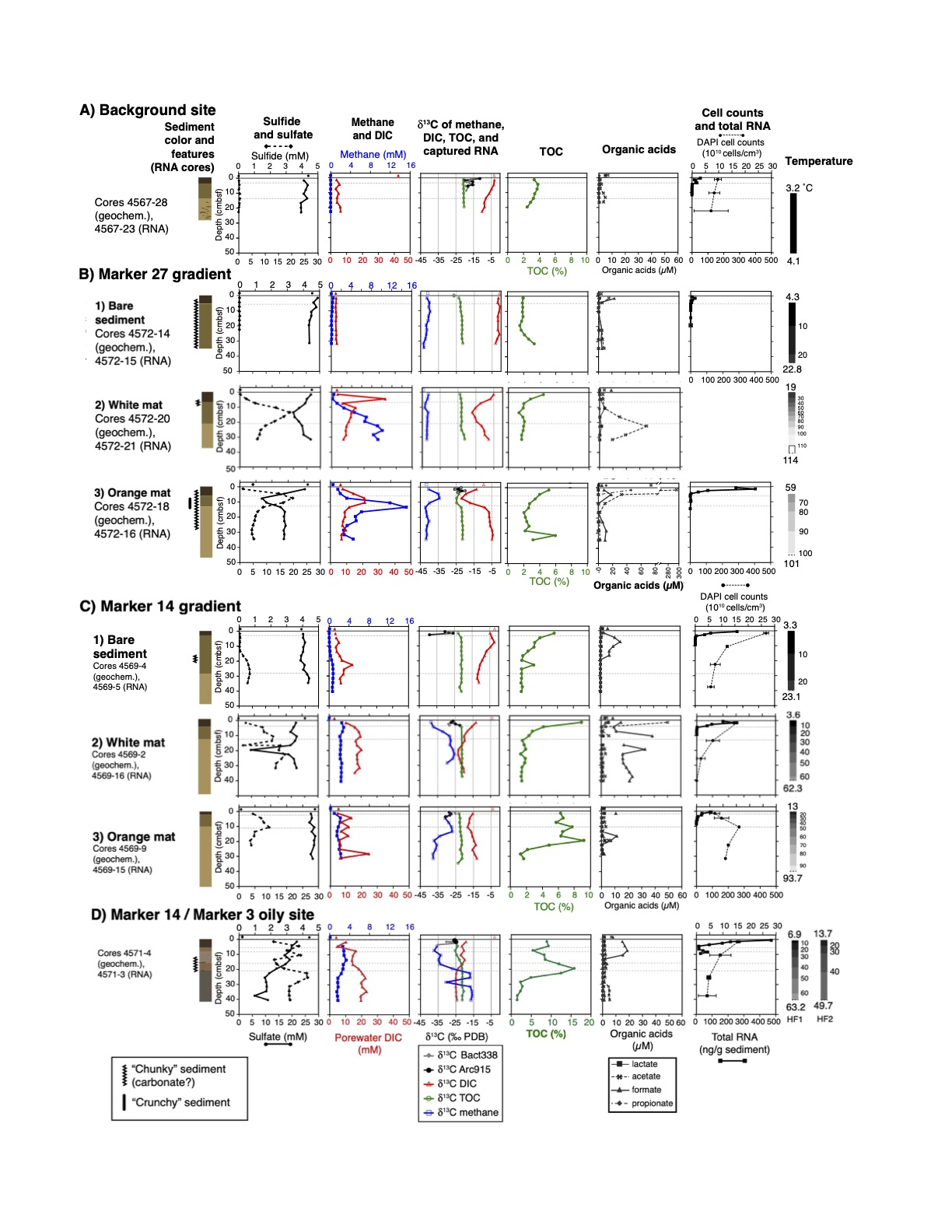
Figure S1**. Biogeochemical profiles of sediment cores extended to 40 cm depth for maximum depth range, in contrast to near-surface resolution within the upper 12 cm as plotted in Figure 2.

**Figure S2**. Biogeochemical and rRNA profiles of sediment cores from sampling sites outside of microbial mat gradients, corresponding to panels C (INSINC cores), F (Marker 5), G (Megamat) and H (Profiler mat) in Figure 1.

**References**

Dombrowski, N., Teske, A.P, and Baker, B.J. (2018). Extensive microbial metabolic diversity and redundancy in Guaymas Basin hydrothermal sediments. *Nature* *Communications* 9:4999; doi: 10.1038/s41467-018-07418-0.

Holler, T. Widdel, F., Knittel, K., Amann, R., Kellermann, M.Y., Hinrichs, K.-U., Teske, A., Boetius, A., and Wegener, G. (2011). Thermophilic anaerobic oxidation of methane by marine microbial consortia. *ISME J.* 5, 1946-1956.

Lin, Y.-S., Koch, B. P., Feseker, T., Ziervogel, K., Goldhammer, T., Schmidt, F., Witt, M., Kellermann, M., Zabel, M., Teske, A., and Hinrichs, K.-U. (2017). Near-surface Heating of Young Rift Sediment Causes Mass Production and Discharge of Reactive Dissolved Organic Matter. *Scientific Reports* 7, 44864; doi:10.1038/srep44864.

Laso-Pérez, R., Wegener, G., Knittel, K., Widdel, F., Harding, K.J., Krukenberg, V., Meier, D.V., Richter, M., Tegetmeyer, H.E., Riedel, D., Richnow, H.-H., Adrian, L., Reetsma, T., Lechtenfeld, O.J., and Musat, F. (2016). Thermophilic archaea activate butane via alkyl-coenzyme M formation. *Nature* 539, 396–401.

McKay, L.J., MacGregor, B.J., Biddle, J.F., Mendlovitz, H.P., Hoer, D., Lipp, J.S., Lloyd, K.G., and Teske, A.P. (2012). Spatial heterogeneity and underlying geochemistry of phylogenetically diverse orange and white *Beggiatoa* mats in Guaymas Basin hydrothermal sediments. *Deep-Sea Research I*, 67:21-31.

McKay, L., Klokman, V., Mendlovitz, H., LaRowe, D., Zabel, M., Hoer, D., Albert, D., de Beer, D., Amend, J., and Teske, A. 2016. Thermal and geochemical influences on microbial biogeography in the hydrothermal sediments of Guaymas Basin. *Environ. Microbiol. Reps*. 8, 150-161.

Pearson, A., Seewald, J.S., and Eglinton, T.I. (2005) Bacterial incorporation of relict carbon in the hydrothermal environment of Guaymas Basin. *Geochim. Cosmochim. Acta* 69, 5477–5486.

Song, M., Schubotz, F., Kellermann, M.Y., Hansen, C.T., Bach, W., Teske, A., and Hinrichs, K.-U. (2021). Formation of ethane and propane via abiotic reductive conversion of acetic acid in hydrothermal sediments. *Proc. Natl. Acad. Sci. USA* 118, e2005219118. <https://doi.org/10.1073/pnas.2005219118>

Teske, A., de Beer, D., McKay, L., Tivey, M.K., Biddle, J.F., Hoer, D., Lloyd, K.G., Lever, M.A., Røy, H., Albert, D.B., Mendlovitz, H., and MacGregor, B.J. (2016). The Guaymas Basin hiking guide to hydrothermal mounds, chimneys and microbial mats: complex seafloor expressions of subsurface hydrothermal circulation. *Front. Microbiol*. 7, 75, doi: 10.3389/fmicb.2016.00075.

Winkel, M., De Beer, D., Lavik, G., Peplies, J., and Mussmann, M. (2014). Close association of active nitrifyers with *Beggiatoa* mats covering deep-sea hydrothermal sediments. *Environ. Microbiol.* 16, 1612–1626. doi: 10.1111/ 1462- 2920.12316
